# African swine fever virus vaccine strain Asfv-G-∆I177l reverts to virulence and negatively affects reproductive performance

**DOI:** 10.1038/s41541-025-01099-9

**Published:** 2025-03-06

**Authors:** Erwin van den Born, Ferenc Olasz, István Mészáros, Eszter Göltl, Barbara Oláh, Jui Joshi, Emma van Kilsdonk, Ruud Segers, Zoltán Zádori

**Affiliations:** 1 MSD Animal Health, Boxmeer, The Netherlands; 2 HUN-REN Veterinary Medical Research Institute (VMRI), Hungária krt. 21, 1143 Budapest, Hungary; 3 Avirtransvac kft, Budapest, Hungary

**Keywords:** Virology, Microbiology, Vaccines, Live attenuated vaccines

## Abstract

ASFV-G-ΔI177L is a modified-live African swine fever virus (ASFV) strain that has been incorporated into a commercially available vaccine. Its safety in pregnant sows and genetic stability in an in vivo passaging experiment were investigated. Upon inoculation of two pregnant sows with ASFV-G-ΔI177L, one developed moderate ASF-related clinical signs. In terms of reproductive performance, 43% of the offspring was born dead and the live-born piglets developed ASF-specific clinical signs, became viremic, and only 17% survived until the end of study. During passaging in pigs, ASFV-G-ΔI177L reverted to virulence with severe ASF-specific clinical signs at passages 3 and 4, associated with increased viremia. Whole genome sequencing identified C257L mutations as a potential driver of increased replication fitness and virulence. The data show that ASFV-G-ΔI177L is not genetically stable and, therefore not safe for use in ASF vaccines and suggest that ASF vaccine candidates should be tested for safety in pregnant animals.

## Introduction

African swine fever (ASF) is a highly infectious and severe haemorrhagic viral disease of swine, causing high mortality in domestic pigs and wild boar. The disease is present on the African, European and Asian continents, as well as in Dominican Republic and Haiti. Outside Africa most viruses are related to the Georgia 2007/1 isolate, commonly referred to as ASFV Georgia 2007 or ASFV-G. This genotype II virus has decimated pig populations in several Asian countries, including China, since 2018. The spread of the virus continues, exacerbated by the fact that in many of the affected regions, the majority of pig production is in backyard and semi-industrial farms with low levels of biosecurity. A number of ASF vaccines have been developed over the past decade, of which the live attenuated virus vaccine strains show the most promise in terms of protection, although they have some inherent safety concerns^1^.

ASFV-G-ΔI177L is a live-attenuated recombinant ASFV vaccine strain that was created by partially deleting the I177L gene and replacing it with the mCherry reporter gene^2^. Commercial pigs vaccinated via the intramuscular or oronasal route were protected against ASFV infection^2–4^. ASFV-G-ΔI177L was shown to be safe and remained genetically stable and phenotypically attenuated during a five-passage reversion to virulence study in domestic swine^5^. However, in 2022, the use of ASFV-G-ΔI177L in a commercial vaccine was associated with the occurrence of serious animal health problems in Vietnam, including mass mortality of pigs. Following the vaccine’s suspension (https://www.reuters.com/world/asia-pacific/vietnam-suspends-african-swine-fever-vaccine-after-pig-deaths-2022-08-24/), Vietnamese health officials concluded that the problem was caused by non-compliance with vaccination guidelines (https://euromeatnews.com/Article-Vietnam-starts-a-new-attempt-of-vaccinations-against-ASF/5752)^6^. Our studies reported in this communication sought to answer two main questions: first, whether the vaccine strain is safe in pregnant sows, and second whether ASFV-G-ΔI177L is genetically and phenotypically stable or whether it regains the functionality of the deleted gene and thus becomes virulent again during in vivo passaging.

The data from our experiments strongly suggest that ASFV-G-ΔI177L cannot be safely used as a modified-live vaccine in the field.

## Results

### Study 1—ASFV-G-ΔI177L immunization of pregnant sows negatively affects their offspring

**Fig. 1:**
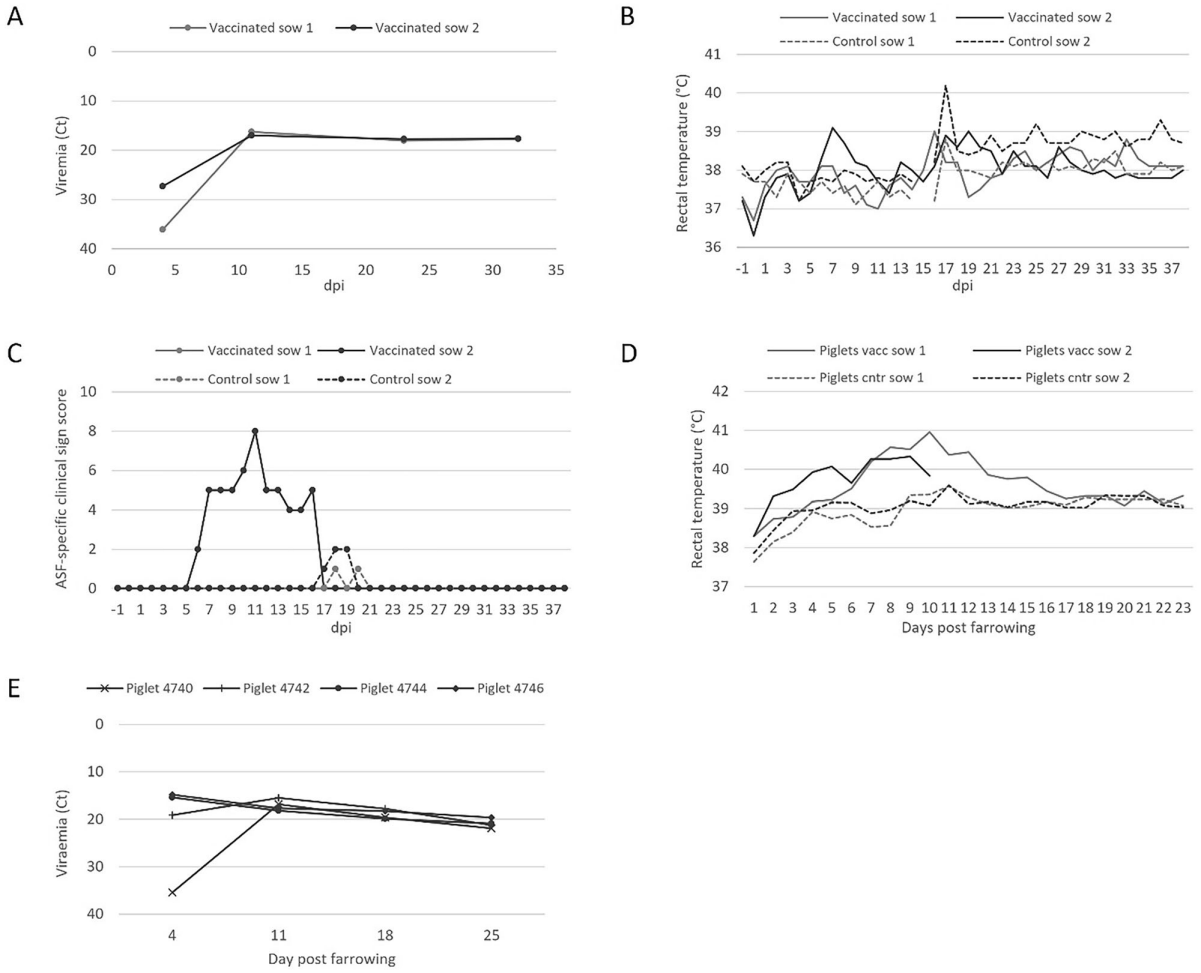
Pre- and post-farrowing data of the pregnant sow study. **A** viremia in ASFV-G-ΔI177L-inoculated sows (group I) presented as ASFV-specific Ct values plotted as a function of dpi. **B** Rectal temperatures of the group I sows and the PBS-inoculated control sows (group II). **C** The sum of ASF-specific clinical signs scores recorded for the sows in both groups. **D** average rectal temperatures of piglets born from groups I and II sows. **E** viremia in the four surviving piglets born in group I presented as ASFV-specific Ct values plotted as a function of days post-farrowing.

To assess the potential risk that the I177L-deleted vaccine strain could pose to pregnant sows and their offspring, two sows in the third trimester of their pregnancy were inoculated with 1.5 × 10^2^ TCID_50_ of ASFV-G-ΔI177L. This inoculation dose corresponded to the lowest effective and safe dose described for this vaccine strain^2^. Both inoculated sows (group I) were viremic on day 4 post-inoculation and remained viremic until the end of the study (Fig. 1A). They also showed blue discolouration around the nipples at 9 dpi. Inoculated sow 1 did not show any other clinical signs, whereas the other inoculated sow (sow 2) had elevated temperatures, was largely inactive, and did not drink and eat on days 7 and 8 post-inoculation (Fig. 1B and C). By day 11, she was still eating less and moving slowly, but by day 14, she was showing signs of recovery with rectal temperatures back to normal, and by day 17, she fully recovered. The two control sows (group II) did not display any ASF-specific clinical signs before and after farrowing, but between 1 and 4 days post-farrowing both control sows were eating less, resulting in a low level of clinical scores (Fig. 1C).

All sows were farrowed at the expected farrowing date at approximately day 115 in gestation and about 15 days post-inoculation. However, significant differences were observed in terms of reproductive performance between inoculated and control sows (Table 1). In the case of inoculated sows, 43% of the piglets were stillborn as compared to 17% of the controls. While all live-born piglets of the control sows survived and were healthy until the end of the study, only 4 of the 23 live-born piglets of the ASFV-G-ΔI177L-inoculated sows survived. Only 8 piglets from inoculated sow 1 and 6 piglets from inoculated sow 2 managed to stay in the study beyond the day of birth, of which all showed ASF-specific clinical signs, and many were euthanized on reaching HEP (Table S5). The average daily rectal temperature of these group I piglets was above the normal temperature range for pigs (38.7–40.0 °C) between 7 and 11 days post-farrowing, whereas the temperatures of piglets born to the control sows remained normal (Fig. 1D). The 4 piglets in the inoculated group that survived until the end of the study were viremic at all the blood sampling timepoints: 4, 11, 18, and 25 days post farrowing (Fig. 1E). Also, ASFV-G-ΔI177L could be detected by PCR in several blood samples taken on the day of farrowing from stillborn or weak-born piglets in group I, indicating that the virus can cross the placental barrier and infect foetuses (Table S6).

**Table 1:** Reproductive performance of the ASFV-G-ΔI177L-inoculated and control sows

	ASFV-G-ΔI177L-inoculated sows		Control sows	
	1	2	1	2
Total piglets born	23	17	27	19
Normal piglets	8	6	21	15
Weak piglets	9	0	2	0
Dead piglets	6	10	4	4
Mummified piglets	0	1	0	0
Alive at end of experiment	4	0	23	15

One piglet was selected from ASFV-G-ΔI177L-inoculated sows 1 and 2 (piglets 4745 and 4765, respectively) that did not reach the end of the experiment, and blood samples taken from these piglets at 11 days post-farrowing were used for NGS analysis to determine the ASFV genome sequence they contained. Comparison of these genome sequences with the sequence of ASFV-G-ΔI177L in the inoculum revealed only the usual 1–2 nucleotide ambiguities in two (15k and 17K) of the long G/C homopolymer regions (Table S8). Higher than 5% nucleotide ambiguities were detected only in 3 genes (EP1242L, C717R and B385R) of ASFV in both animals (Table S9).

These results suggest that ASFV-G-ΔI177L can induce fever and clinical symptoms of ASF in pregnant sows and that the virus can cross the placenta to infect the foetus with a significant negative impact on litter survival.

### Study 2—ASFV-G-ΔI177L can revert to virulence within 3 passages in pigs

**Fig. 2:**
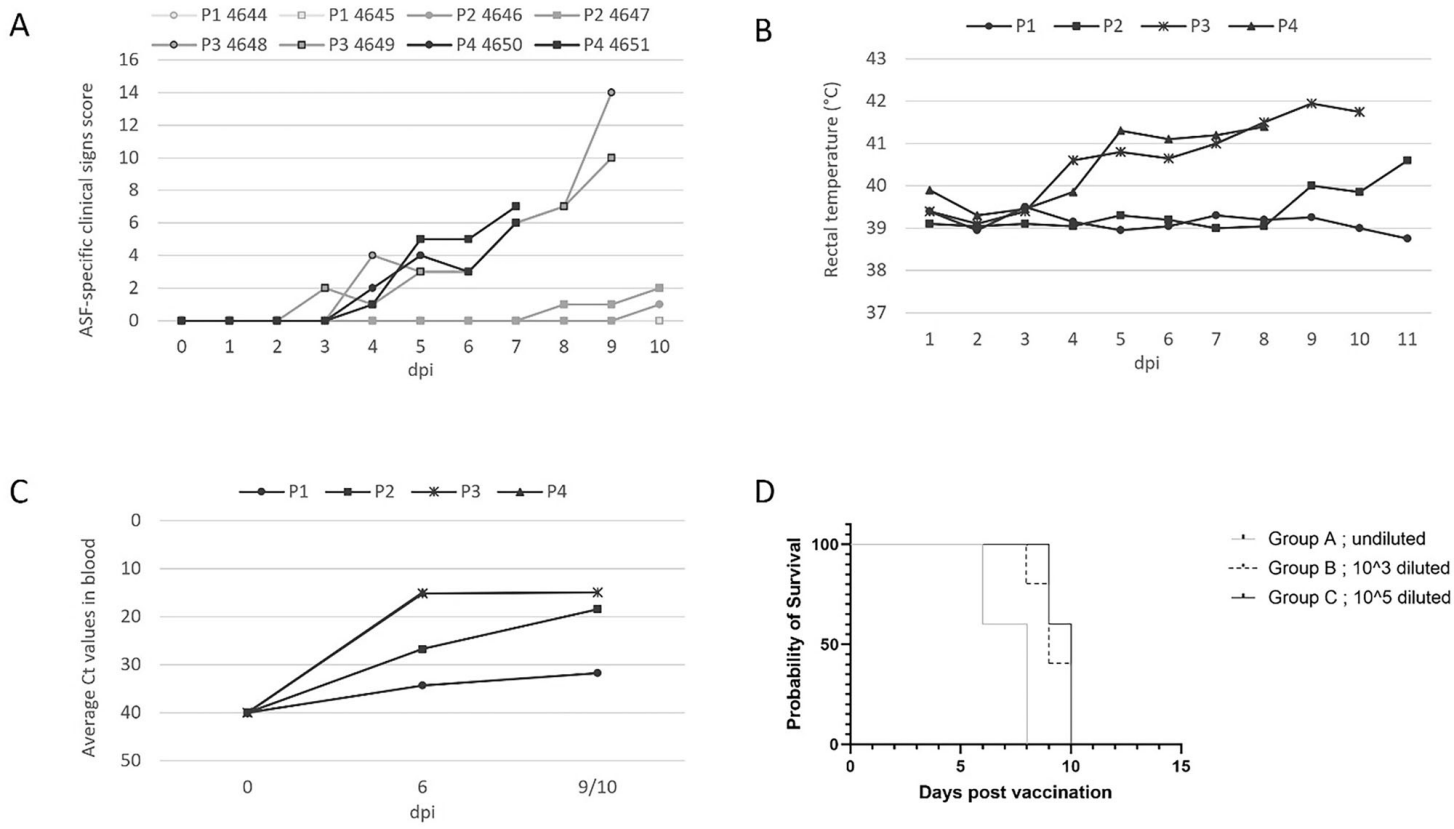
ASFV-G-ΔI177L infection-related data obtained in the reversion to virulence study. P1–P4 indicate the passage level during the in vivo passaging experiment. **A** The sum of the ASF clinical signs scores recorded during each passage. **B** Average rectal temperature; **C** Average level of ASFV viremia in each passage presented as Ct values plotted against dpi. **D** Survival rate of the pigs after inoculation with ASFV-G-ΔI177L P4 virus diluted at different levels.

It has been demonstrated that ASFV-G-ΔI177L sheds from inoculated animals and spreads to naïve comingled animals^5^. Therefore, the virus can transmit through the pig heart, allowing it to adapt to pigs and potentially gain in vivo replication fitness and losing its attenuated phenotype. To evaluate the genetic stability of ASFV-G-ΔI177L, a reversion to virulence study was performed. In passage 1 of this in vivo passaging experiment, two animals (nos. 4644 and 4645) were inoculated with ASFV-G-ΔI177L at a dose of 1.5 × 10^2^ TCID_50_ per animal. Following infection, the two animals did not display any ASF-related clinical signs (Fig. 2A; Table S10), and rectal temperatures remained normal and below 40.0 °C (Fig. 2B).

Pooled blood from animal 4644 taken on 6 and 10 dpi with an average Ct-value of 27.9 was used as inoculum for passage 2 (Table 2b). In the two animals of passage 2, animals 4646 and 4647, elevated rectal temperatures (i.e. above 40.0 °C) were measured from 8 dpi onwards (animal 4647) and 10 dpi (animal 4646). Animals 4647 had sunken flanks at 10 dpi.

**Table 2:** Overview of the animal study designs

(a) Animal study 1—Details of the pregnant sow study				
Group	No. of animals	Inoculum	Inoculation (volume, route)	Inoculation (dose)
I	2	ASFV-G-ΔI177L	2 ml IM, neck	1.5 × 10^2^ TCID_50_
II	2	PBS	2 ml IM, neck	–

(b) Animal study 2—Details of the in vivo passages and the treatment of the animals					
Passage	2 animals	Inoculum	Inoculation		End of passage (dpi)
			Volume, route	Dose	
1	4644 and 4645	ASFV-G-ΔI177L grown in PBMC	1 ml IM, neck	1.5·10^2^ TCID_50_	10
2	4646 and 4647	Pooled blood from (P1) 4644 taken 6 and 10 dpi	1 ml IM, neck	3.0·10^4^ TCID_50_ 5.81·10^4^ GCE	10
3	4648 and 4649	Pooled blood from (P2) animals 4646 and 4647 taken 10 dpi	1 ml IM, neck	3.2·10^5^ TCID_50_ 8.96·10^6^ GCE	9
4	4650 and 4651	Pooled blood from (P3) animals 4648 and 4649 taken 9 dpi	1 ml IM, neck	1.4·10^6^ TCID_50_ 1.64·10^7^ GCE	6

(c) Animal study 3—Details of the different treatment groups				
Group	No. of animals	Inoculum	Inoculation (volume, route)	Inoculation (dose)
A	5	Pooled blood from (P4) animals 4650 and 4651 on day 6 (undiluted)	1 ml IM, neck	1.4 × 10^7^ TCID_50_ 3.08 × 10^7^ GCE
B	5	Pooled blood from (P4) animals 4650 and 4651 on day 6 (1000x diluted in PBS)	1 ml IM, neck	1.4 × 10^4^ TCID_50_ 3.23 × 10^4^ GCE
C	5	Pooled blood from (P4) animals 4650 and 4651 on day 6 (100,000x diluted in PBS)	1 ml IM, neck	1.4 × 10^2^ TCID_50_ 5.27 × 10^2^ GCE

Dose is expressed in 50% tissue culture infectious doses (TCID50) and genome copy equivalents (GCE).

Pooled blood from animals 4646 and 4647 taken on 10 dpi with an average Ct-value of 18.5 was used as inoculum for passage 3 (Table 2b). The two animals of passage 3 (animals 4648 and 4649) showed ASF-related clinical signs starting on day 3 post-inoculation, and one of the animals had a rectal temperature above 41.0 °C (Fig. 2; Table S10). At 4 dpi both animals had an elevated rectal temperature (i.e., above 40.0 °C), and one of the animals was slow and ate less. One and two days later, at 5 and 6 dpi, both animals were slow, ate less, and had elevated rectal temperatures. At 7 dpi, the ASF-related clinical signs worsened, as the animals ate little to nothing at all, had sunken flanks, were slow, and their rectal temperatures were above 41.0 °C. One day later, the ASF-related clinical signs remained the same, the posture of the animals was stiff, and the back was arched when standing up. At 9 dpi, the ASF-related clinical signs got even worse. On top of the observation at 8 dpi, the back of one of the animals (number 4648) remained arched when walking around, and this animal was slimming. Both animals were slow and got up with some help but lay down quickly. Both animals developed neurological signs such as somewhat doubtful gait, deviant leg position, which was slowly corrected (animal 4648) and ataxia or weakness of the hindquarters but still able to walk (animal 4649). One of the animals (animal 4648) had diarrhoea. At this point, both animals reached HEP and were euthanised.

Pooled blood from animals 4648 and 4649 (P3) taken on 9 dpi with an average Ct-value of 14.9 was used as inoculum for passage 4 (Table 2b). At 3 dpi, one of the animals, 4650, had a shaky motion, and both animals started to increase their rectal temperature from 4 dpi onwards (Fig. 2A; Table S10). One day later, the animals ate little to nothing at all and were slow. At 6 dpi, both animals had rectal temperatures above 41.0 °C one of the animals (4650) ate slowly, and the other animal (4651) ate little to nothing at all and had sunken flanks. At 7 dpi the ASF-related clinical signs worsened, as the animals ate little to nothing at all, the animals had sunken flanks, were slow, and the rectal temperatures were above 41.0 °C. At this point it was decided to euthanise the animals for animal welfare reasons, and the scientific endpoint of the study had been reached.

Viremia in passages 1 and 2 animals was measured at 6 and 10 dpi, while for passage 3 animals it was measured at 9 dpi instead of 10 dpi, and only at 6 dpi for passage 4 animals (Fig. 2C). At 6 dpi one of the animals of passage 1 was viremic, and at 10 dpi ASFV DNA was detected in both animals at an average Ct of 31.7. In passage 2, the animals were viremic at 6 dpi, and viremia was increased at 10 dpi to an average Ct of 18.5, which is about 10,000 times more than measured in passage 1 animals. In passage 3, the animals were viremic at 6 dpi and viremia was increased at 9 dpi to an average of 14.9 Ct, which is again approximately 10-fold higher than measured in the animals of passage 2. The level of viremia at 6 dpi in passage 4 was very similar to that in passage 3. A later blood sample from the passage 4 pigs was not taken as the passage was prematurely aborted.

### Study 3—Safety in pigs of diluted blood of passage 4

The blood samples in Study 2 were used undiluted as inoculum, and therefore, animals in passage 3 received approximately 2000 times (1.5 × 10^2^ vs. 3.18 × 10^5^ TCID_50_) more ASFV compared to the animals in passage 1 (Table 2b). Likewise, the animals of passage 4 received approximately 5 times more ASFV compared to the animals of passage 3 (3.18 × 10^5^ vs. 1.46 × 10^6^ TCID_50_). It is commonly known in the ASFV field that animals inoculated with a high virus dose can display an earlier onset of and/or higher viremia than animals inoculated with a lower virus dose. Also, the observed virulence of an ASFV strain can depend on the dose, with enhanced virulence at higher doses. To rule out that the increased virulence observed in the in vivo passaging study is (partially) caused by the fact that animals in passages 2–4 received a high dose of ASFV-G-ΔI177L, a follow-up study was conducted in which animals were inoculated with passage 4 blood samples that were undiluted or 10^3^ and 10^5^ times diluted, thereby this follow up study represented passage 5.

The infectious titre of the 100,000×-diluted inoculum was 1.4 × 10^2^ (Table 2c), which is almost exactly the same (1.5 × 10^2^) as the inoculum used for passage 1 in Study 2 (Table 2b). Moreover, these doses are well within the described safety range of ASFV-G-ΔI177L^2^. Since the animals in passage 1 showed no clinical signs, the same could be expected for the 100,000× diluted group if the virus did not change genetically.

However, regardless of the dose of the 4-times-passaged ASFV-G-ΔI177L strain (‘P4 virus’), none of the animals survived in the study (Fig. 2D), and all of them displayed severe clinical signs (Table S7). It was observed that a shorter onset of clinical disease and earlier mortality after inoculation correlated with a higher dose, supporting the notion that a higher viral dose increases virulence. A high level of viremia was detected at 7 dpi with Ct values between 11.9 and 13.5 and values did not differ between the groups (Table S7). This data confirmed that P4 virus was virulent, and the high inoculation dose cannot be responsible alone for the high level of viremia noticed in the reversion to virulence study (Study 2).

### Genetic analysis of in vivo passaged ASFV-G-ΔI177L

The results of Studies 2 and 3 demonstrated that ASFV-G-ΔI177L reverted to virulence and gained replication fitness during in vivo passaging, suggesting that sequence alterations have occurred in the virus genome. To investigate this possibility, the genome of the virus obtained from passage 3 in Study 2 (animal 4648) was directly sequenced (i.e. without prior isolation of the virus in tissue culture), because ASF-related clinical signs became first explicit during this passage. Comparison of the P3 virus sequence with the original (P0) sequence revealed differences in the poly G/C stretches of the MGF 110-11L and MGF 110-14L genes in the form of nucleotide deletions or insertions, as well as differences in the form of polymorphic positions limited to the C257L and B385R genes (Tables S11a and S12).

To get a complete picture of the changes that occurred during passaging, these regions were PCR amplified, including all the longer poly G/C stretches, from viruses of each passage, and sequenced the amplicons (Fig. S1). Comparing the size of the poly G/C stretches of the passaged viruses with that of the P0 virus showed that they occasionally diverged 1-2 nucleotides in the different passages but without an obvious direction (Table S8). Mutations or polymorphisms were not detected in the transgenic region, demonstrating that the sequence of the transgene (mCherry regulated by the p72 promoter) remained stable throughout the passages (Table S12). The B385R ORF exhibited a polymorphic nucleotide at position 104908 (cytosine/thymine) in passages 2 and 3 but reverted to its original cytosine form and became homogenous again in passage 4 (Tables S11, S12).

**Fig. 3:**
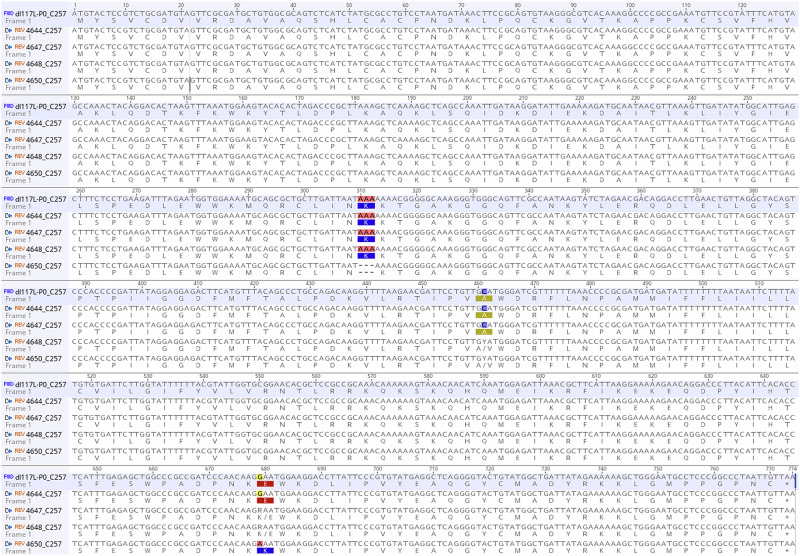
Alignment of the C257L sequence identified in the different passages. P1 animal 4644; P2 animal 4647, P3 animal 4648, P4 animal 4650.

The only changes showing some successions were detected in three separate nucleotide positions in the C257L gene. At position 86868, cytosine became polymorphic (cytosine/thymine) in passages 2 and 3, and subsequently changed to thymine in passage 4, resulting in an amino acid change from glutamic acid to lysine. The C257L ORF contains a homopolymer stretch of seven thymine residues at nucleotide position 87231–87237, which was reduced to four thymines in passage 4, causing a deletion of one lysine residue in the C257L protein. At position 87086, guanine exhibited polymorphism (guanine/adenine) in passages 3 and 4. In the late passages, the transition from guanine to adenine resulted in an alanine to valine amino acid substitution (Tables S11, S12, Fig. 3). NGS sequencing of the full genome of passage 4 virus confirmed the sequencing results of the PCR amplicons, and no other additional mutations were found in the virus genome. To determine the relative position of the three SNPs in the virus quasispecies, the C257L gene was PCR amplified and cloned. From the resulting clones, 5 were sequenced. Four out of the five clones carried the C86868T and the three thymine deletions at position 87231–87237 in combination, while the remaining clone carried only the latter, and the adenine mutation at position 87086 could not be detected in any of the clones.

These results suggested that mutations in the C257L protein might have played a major role in the observed reversion to virulence of ASFV-G-ΔI177L. Additional in silico and in vitro experiments were performed to obtain further data to support this hypothesis. Examination of all the ASFV C257L genes (365) available in GenBank, representing sequences from many ASFV genotypes, revealed that the C257L gene is highly conserved at the protein level, and no natural or engineered ASFV genome contains the lysine deletion and the glutamine-lysine substitution at amino acid positions 105 and 227 of the C257L gene, respectively, and the valine is also very rare at position 154 and characteristic for some XV and VIII ASFV genotypes (ON409981.1 and NC_044954) (Fig. S2).

To study the effect of the identified C257L mutations in another attenuated ASFV strain, modification of the C257L gene of the ASFV-G-Δ9GL/ΔUK virus^7^ was attempted by introducing the aforementioned mutations. For this, first the C257L gene had to be knocked out by inserting an eGFP reporter gene under the control of the p72 promoter. For the design of the Crispr/Cas9-mediated homologous recombination transfer plasmid, it was considered that the transcripts of the neighbouring C122R and C475L genes terminate in the C257L gene^8,9^, which limits the size of the C257L gene sequence that can be deleted without the potential functional loss of the product of the neighbouring genes. Despite careful design, a C257L knock-out virus could not be rescued after several independent attempts, which suggests that the C257L gene is essential for ASFV.

## Discussion

To obtain market authorization for a new ASF vaccine, like one based on the ASFV-G-ΔI177L strain, its safety must be thoroughly demonstrated in animal studies. Such testing is particularly important for live virus vaccines that can be shed by vaccinated animals at some point in the post-vaccination phase and spread to susceptible (naïve) animals. Our experiments revealed that ASFV-G-ΔI177L infection could induce ASF clinical symptoms, including fever in pregnant sows, and that the virus is able to cross the placenta and replicate in the foetus leading to low survival rates in foetuses and piglets. As ASFV-G-ΔI177L-vaccinated animals were reported to shed the virus resulting in its spread to neighbouring unvaccinated animals^5^, our finding suggests that the use of the ASFV-G-ΔI177L strain during mass vaccination could pose an unacceptably high risk to pregnant sows, even if they are not vaccinated themselves. It is of particular concern that the virus replicates at high titres in the foetus, and therefore, large amounts of the virus can be released into the environment when a vaccinated sow gives birth to infected foetuses, significantly increasing the chances of super-dose virus infection of nearby animals and spreading the virus to distant areas.

In the pregnant sow study, a group size of 2 animals does not allow statistical analysis with adequate power. However, the small group size was chosen considering animal welfare and space limitations in housing pregnant animals and their litters in high containment units. Follow-up studies with larger group sizes are required to determine the proportion of ASFV-G-ΔI177L- vaccinated sows displaying reproductive issues. Nevertheless, the fact that both inoculated sows showed such a profound negative effect should be of great concern, knowing that sows are sometimes vaccinated in the field although not recommended by the product label and that ASFV-G-ΔI177L could potentially spread from vaccinated pigs to unvaccinated (naïve) pregnant sows^5^.

Differences between the genomes of the virus used to inoculate the pregnant sows and viruses isolated from the born piglets are limited to 1–3 single nucleotide polymorphisms detected in three genes with potentially high biological impact: EP1242L (DNA-dependent RNA polymerase subunit 2)^10^ C717R (protein with serine/threonine kinases domain, which stimulates inflammation)^11^ B385R (late transcription factor-like Zinc finger protein)^12^, and two poly C/G regions. As none of the newly emerged nucleotide variants of the three high-impact genes are dominant in the virus pool of the piglets and poly C/G regions are notoriously difficult to sequence and their importance in the virulence is unknown, we do not wish to speculate about the effect of this mutation on placental crossing or piglet infection. However, it is abundantly clear that the negative effect on the reproductive performance of sows is an intrinsic feature of ASFV-G-ΔI177L, regardless of whether the significance of the viral nucleotide polymorphism in this process is known precisely.

The results of the in vivo passaging experiment demonstrated that ASFV-G-ΔI177L reverted to virulence. The marked increase of ASFV viremia upon passaging suggests that ASFV-G-ΔI177L gained replication fitness, which correlates with an increase in ASF-related clinical signs and rectal temperature. After four in vivo passages, mutations in the C257L gene occurred in addition to the usual changes in the poly C/G regions. C257L is a putative structural protein with an unknown function and a single transmembrane segment^13,14^. Two of the three C257L mutations do not occur in any published ASFV strain, which may suggest that they are compensatory mutations that facilitate viral infection in the absence of the I177L protein. This assumption is further strengthened by the fact that, although the exact function of neither the I177L protein nor the C257L protein is known, both are present in the virion^13^. Our unsuccessful attempts to rescue a C257L knock-out mutant virus strongly suggest that the C257L protein is essential to complete the ASFV life cycle.

Contrary to our results, ASFV-G-ΔI177L was reported to be genetically stable and did not revert to virulence in a passaging experiment similar to the one presented here^5^. However, looking closer at the evolution of the titres during in vivo passaging presented in the publication of Tran and co-workers, the viremia in passage 5 animals was significantly higher and its duration longer than in passage 1. Remarkably, whole genome sequencing revealed that the P5 virus, as compared to the original inoculum, had a G-to-A nucleotide change at position 87056 that occurred in 99% of the sequencing reads, changing a methionine to a threonine in the C257L protein. Whether this amino acid change increased the replication fitness of ASFV-G-ΔI177L without resulting in increased virulence is unclear.

In summary, it can be concluded from our work that ASFV-G-ΔI177L is both genotypically and phenotypically unstable during horizontal or vertical transmission and is therefore an unsafe vaccine strain.

## Materials and methods

### ASFV strain

The ASFV-G-ΔI177L strain was obtained under license no. 1861-002 from Agricultural Research Service (ARS), USDA. After the strain was generated by ARS^2^, they propagated it on primary porcine blood macrophages (PBMC). The stock received was not further amplified (i.e., used directly in animal studies 1 and 2) and had a virus titre of 3.2 × 10^4^ TCID_50_/ml. TCID_50_ was determined in all experiments by FLI IFA method^14^ by performing 10× serial dilutions of the stocks in microtiter plates containing porcine alveolar macrophages (PAM) and scoring the cytopathic effect. Titres were calculated using the Reed–Muench method^15,16^. It is worthwhile noting that ASFV titres expressed as TCID_50_ correspond well with titres expressed as HAD50^15^ used by Borca et al.^2^.

### Pigs

Male pigs of 6–9 weeks of age when inoculated and pregnant sows at approximately 100 days in gestation at the time of inoculation were selected from the same farm with high health status. The breeds of the pigs were TLLZZ (Topigs Norsvin 70 (TN70); Large White (♀) × Norsvin Landrace (♂)) and TZZZZ (Large White (♀) × Large White (♂)), which are very common in Europe. All pigs were negative for antibodies against ASFV and negative for ASFV at the time of inoculation. They were also negative for Porcine respiratory and reproductive syndrome virus (PRRSV). The pregnant sows were Porcine parvovirus 1 (PPV1) negative and vaccinated against PPV1, and their age and reproductive history are summarized in Table S1.

### Animal housing and compliance

All animals were housed in the high containment facility of Wageningen Bioveterinary Research (WBVR, Lelystad, The Netherlands). Housing complied with European Directive 2010-63 on the protection of animals used for scientific purposes (https://eur-lex.europa.eu/eli/dir/2010/63/oj). Tap water was available ad libitum. Pigs were given commercial pig feed. Sows were fed with commercial sow feed, and their piglets received colostrum and milk from their mother.

During all treatments, animals were conscious and not anaesthetised. Animals that reached a humane endpoint (HEP) and all remaining animals at the end of each study were euthanized by intravenous administration of 0.5 ml/kg pentobarbital (Euthasol 20%; AST Farma B.V.) after sedation with an intramuscular injection of a mixture of tiletamide-zolazepam (Zoletil 100; Virbac) and xylazine (Sedanum; Eurovet Animal Health B.V.) at 0.1 ml/kg.

Each animal trial was performed in compliance with the Dutch law on Animal Experiments under project license number AVD22100202114853 obtained from Centrale Commissie Dierproeven. In addition, the protocol of each animal trial was checked against the license and animal welfare criteria by the Animal Welfare Bodies of both MSD Animal Health and WBVR.

### Animal study 1—Safety in pregnant sows

Four pregnant multiparous sows were available for this study. At around 100 days in gestation, two sows received ASFV-G-ΔI177L in the neck muscle at a dose of 1.5 × 10^2^ TCID_50_ diluted in 2 ml PBS, while two other sows served as controls receiving 2 ml of PBS. Animals were monitored from inoculation onwards for ASF-specific clinical signs. Whole blood (EDTA) samples were collected at 4, 11, 23, and 32 days post inoculation (dpi) and analysed for the presence of ASFV by qPCR. Upon birth, the health status of the piglets was monitored and recorded. Piglets were monitored daily for ASF-specific clinical signs until the end of the study. Table 2a provides an overview of the study design and treatment.

### Animal study 2—Reversion to virulence

A reversion to virulence study was performed according to European Pharmacopoeia section 5.2.6. ‘Evaluation of safety of veterinary vaccines and immunosera’ (https://www.edqm.eu/en/european-pharmacopoeia-ph.-eur.-11th-edition) and VICH guideline 41 ‘Guideline of target animal safety: examination of live veterinary vaccines in target animals for absence of reversion to virulence’ (https://vichsec.org/guidelines/). ASFV-G-ΔI177L was passaged four times in two 7- to 9-week-old pigs (a minimum of 2 animals is required for the first 4 passages) at the time of inoculation (Table 2b). The required passage 5 with a minimum of eight animals was cancelled due to the development of severe clinical signs in animals of passages 3 and 4 and was substituted by “Animal study 3”, which was carried out with fifteen animals. Of note, the age of the animals is in line with the recommended youngest age for NAVET-ASFVAC (i.e. 8–10 weeks), a commercial ASF vaccine containing strain ASFV-G-ΔI177L.

The two pigs available for passage 1 (nos. 4644 and 4645) received each 1 ml of virus inoculum (1.5 × 10^2^ TCID_50_/ml) in the neck muscle. From the day of inoculation, the animals were observed daily for ASF-related clinical signs for 10 consecutive days (or shorter in case an animal had to be euthanized before the end of the passage due to reaching a HEP). Whole blood (EDTA) was drawn just before inoculation, at 6 dpi, and at 10 dpi (or earlier in case of HEP), and analysed for the presence of ASFV by qPCR. The two blood samples with the highest amount of ASFV DNA were pooled and the pool was used as inoculum for the next passage, similar to as described previously^5,17^.

The two pigs available for passages 2 (animals 4646, 4647), 3 (animals 4648, 4649), and 4 (4650 4651) were inoculated intramuscularly (IM) in the neck muscle with 1 ml of undiluted pooled blood generated from the two blood samples with the highest amount of ASFV DNA obtained in the previous passage. Blood sampling and observation of clinical signs were as described for passage 1.

### Animal study 3—Confirmation of virulence of in vivo passaged ASFV-G-ΔI177L

Fifteen male pigs, 6-week-old at the time of inoculation, were available for this experiment. The 5 animals in group A received via the neck muscle 1 ml of undiluted pooled blood collected on 6 dpi from animals 4650 and 4651 in passage 4 of Study 2, while the 5 animals in group B received 1 ml of this blood 1000× diluted (in PBS) and the 5 animals in group C received 1 ml of 100,000× diluted blood (Table 2c). EDTA blood samples were taken just before inoculation and weekly thereafter and investigated for the presence of ASFV by PCR. After inoculation, animals were observed daily for ASF-specific clinical signs.

### Scoring of ASF-related clinical signs

The following ASF-related clinical signs were scored: posture, body shape, appetite, activity, rectal temperature, vomiting, breathing, neurological signs, skin colour, and secretions (nasal, ocular, and rectal). For each ASF-related clinical signs parameter, a score of 0, 1, 2 or 3 was given (Table S2). HEP was reached if the sum of the ASF-related clinical score reached 10 or above. The highest score (3) for the parameters posture, activity, vomiting, breathing, neurological signs or secretions was also the reason for HEP. As part of the ASF-related clinical signs, rectal temperature was measured just before inoculation and daily thereafter.

### ASFV qPCR

EDTA blood samples were analysed by qPCR for the presence of ASFV DNA. ASFV DNA was extracted from EDTA blood samples (100 μl EDTA-blood + 100 μl PBS per sample) using the MagNA Pure 96 DNA and Viral NA Small Volume Kit on a MagNA Pure 96 (Roche Diagnostics). The extracted DNA was analysed for the presence of ASFV DNA using the qPCR method of the ASF National Reference Laboratory of The Netherlands (WBVR)^15^. Samples that crossed the threshold, were counted as positive, and the corresponding Ct-value was reported. Samples that did not cross the threshold within 45 cycles were considered negative and a Ct value of 45.0 was used for calculations. Absolute genome copy numbers were calculated using a standard curve as described by Olesen et al.^15^.

### Sequencing and sequence analysis

The full-length genome sequence of ASFV-G-∆I177L was obtained through next-generation sequencing (NGS). DNA preparation was performed as described earlier^18^, directly from blood samples without amplification of the virus in cell culture. Purified ASFV DNA was sent for sequencing to Novogene Co. where the sequence was determined using Illumina Novaseq X Plus. For each genome over 12 million 150 bp paired end reads were assembled (Table S3) using Geneious Prime 2019.2.3 (https://www.geneious.com/).

For analysis, the Bowtie2 mapping tool was used with normal sensitivity. As a template the ASFV-G-ΔI177L sequence was used provided by the Agricultural Research Service (ARS); USDA extended at the 3’ and 5’ ends with the sequence of the reference strain ASFV/Timor-Leste/2019/1 (accession number: MW396979). Nucleotide numbers in the genome of each passage correspond to positions in the original genome in the inoculum. (Acc. number: PV067679). The row sequence data of the project are deposited in the BioProject database (ID: PRJNA1218536).

Five homopolymer regions (i.e. poly-G and poly-C stretches), the transgenic area (i.e. where the P72-mCherry cassette was inserted in the I177L gene), and two genes (i.e. C257L and B385R) (Fig. S1) were amplified using the Primestar GXL kit with GC buffer (Takara Bio Inc., Japan) in a 25 μl final volume with 1 μl of purified ASFV DNA, according to the manufacturer’s instructions. The primer pairs used for the amplification of the different fragments are listed in Table S4. The amplicons were sequenced by either the Sanger method from both directions or by NGS. The size and sequence of the poly-C and poly-G stretches were determined by sequence alignments using Geneious Prime 2019.2.3.

## Supplementary information

Supplementary datas

## Data Availability

The row sequence data of the project are deposited in the NCBI BioProject database (ID: PRJNA1218536). Any additional data supporting the results can be found in the publication or in the Supporting Information file.
