# Supplementary material for: African swine fever virus vaccine strain Asfv-G-∆I177l reverts to virulence and negatively affects reproductive performance
Source: NPJ Vaccines. 2025 Mar 6;10:46. doi: 10.1038/s41541-025-01099-9 (PMC11885574; doi:10.1038/s41541-025-01099-9)

Table S1. Reproductive history details of the sows included in the pregnant sow study

Animal number	Age of sow at farrowing (months)	Previous reproductive performance				
		Birthing cycles	Live piglets	Dead piglets	Total piglets	Average no. piglets per litter
4662 (Vaccinated sow 1)	31	4	76	4 (5%)	80	20
4663 (Vaccinated sow 2)	26	3	37	5 (12%)	42	14
4664 (Control sow 1)	46	7	136	11 (7%)	147	21
4665 (Control sow 2)	46	7	115	5 (4%)	120	17

Table S2. ASF-related clinical scoring list

	**Posture**
0	Normal
1	Stiffness and arched back when standing up, then 'normal'
2	Stiffness and arched back remain when walking around
3 (HEP)	Muscle cramps / cramping of the muscles
	**Body shape**
0	Normal
1	Sunken flanks
2	Slimming
3	Wasting (ribs, spinal vertebrae visible, long hair)
	**Appetite**
0	Normal
1	Slow eater, does eat feed
2	Approaches fed tray, tastes feed, but eats little/nothing
3	Does not eat, no interest in feed
	**Activity**
0	Normal
1	Slow, still gets up on its own without help
2	Slow, gets up with some help, lies down quickly
3 (HEP)	Stays down, doesn't get up even after some pressure
	**Rectal temperature**
0	38.0 - 40.0
1	40.1 - 41.0
2	> 41.0 or <38.0
3	> 41.0 longer than 48 hours
	**Vomiting**
0	Normal
1	Occasional vomiting (1x during observation period)
2	Repeated vomiting (several times during observation period)
3 (HEP)	Bloody vomit
	**Breathing**
0	Normal
1	Increased breathing rate, occasional coughing or sneezing
2	Pumping breathing rate, abdominal breathing, frequent coughing or sneezing
3 (HEP)	Difficult breathing, panting, breathing with open mouth
	**Neurological signs**
0	Normal
1	Somewhat doubtful gait, deviant leg position which is slowly corrected
2	Ataxia/weakness of the hindquarters, can still walk
3 (HEP)	Paralysis, can't stand anymore, not even with some help
	**Skin (particularly on ears, muzzle, tail, legs, abdomen)**
0	Normal
1	Red skin
2	White, blue/purple or other discoloration of the skin, possibly bleedings in skin
3	Large blue/purple spots, large skin hemorrhages, skin necrosis/ulcer
	**Secretions (eye, nose, rectum)**
0	Normal
1	Thin, clear discharges from nose and/or eye (without admixtures) or diarrhoea
2	Thich discharge from nose and/or eyes (puss-like or colored, no blood)
3 (HEP)	Bloody discharge (fresh blood from nose, eyes or rectum) or black manure (old blood)

Table S3. Genome assembly statistics. The lowest coverage was found in the first or last nucleotides of the genomes in all the cases.

Pig and passage no.	Minimum coverage	Maximum coverage	Average coverage	Number of reads
ASFV-G-ΔI177L, P0	125	110 540	11592	13 600 000
4648, P3	9	77 650	18593	14 720 000
4650, P4	19	45 377	10 473	12 385 000
4745	108	240 034	15 049	15 482 000
4765	56	200 133	13112	13 820 000

Table S4. Primers used in this work

Name of target region or gene	Primer name	Primer sequence (5’ to 3’)
14k poly-C (15055-15065)	seqASF_14234-F	CTGAGATAGCCAAATCAAAATAC
	seqASF_14234-R	CGATTGTAAACTGTATAGTTAATCG
15k poly-C (16494-16509)	seqASFV_15670-F	CAAAGCAGCCTGTATATGCAATACC
	seqASFV_15670-R	CAATCATTCTATTGTAAACTGTAGAG
17k poly-G (18450-18464)	PCR_1_F	GGATACACAGGTCTTTAGTCTGC
	PCR_1_R	AGCTTAAACAAGCGACGTCT
	seq17400_F	TTATGAATAACAGGAGTAAACG
	seq17700_R	ACAATTAAGCCTCAGAACTAC
17k poly-G (18670-18680)	PCR_2_F	GAATACTTCATAGATGCTAAACATT
	PCR_2_R	TACGTTTAGATCCAAATGACATG
	seq17700_F	GTAGTTCTGAGGCTTAATTGT
	seq18200_R	GCTGAGAGACAATTTGCGGT
20k poly-G (20826-20839)	seqASFV_20022-F	TAGTACATCAATGTTGTAAGTTTG
	seqASFV_20022-R	CTATCTAAACGTGCTTCTATGAATTC
C257L	seq2_C257L_F	CGCACCAATACGTAAAAAATACC
	C257_fullR	CTTTAGCCCGGCATATAGAATC
B385R	B385_fullF	GTTTCTTCGGATATTTCAGTAGAG
	B385_fullR	TCTGCTATTAACTTTCTTCTTCTTC
pp72-mCherry	SEQendI177L_F	GCGATTTTTGCCTTGAATAAG
	SEQendI177L_R	GGACATCACCTCCCACAACG
	seqI117L_F	CCCTTGCTCACCATATAATGTT
	seqI117L_R	TATTCCACTCTGATACTCCCCAG

Table S5. ASF-specific clinical sign scores and rectal temperatures in viable piglets born from the ASFV-G-ΔI177L-inoculated sows.

Day post farrowing	D0	D1	D2	D3	D4	D5	D6	D7	D8	D9	D10	D11	D12	D13	D14	D15	D16	D17	D18	D19	D20	D21	D22
Piglet ID	**ASF-specific clinical sign scores for piglets in litter of inoculated sow 1 (animal ID 4662)**																						
4740	0	0	0	0	0	0	0	2	1	2	2	2	0	0	0	0	0	0	0	0	0	0	0
4741	0	0	0	0	0	0	1	3	1	2	7												
4742	0	0	0	0	0	0	1	4	1	3	2	2	1	2	3	4	4	3	3	2	2	0	0
4743	0	0	0	0	0	0	1	2	3	3	9												
4744	0	0	0	0	0	2	1	2	1	2	2	1	1	0	0	0	0	0	0	0	0	0	0
4745	0	0	0	0	0	0	0	2	1	2	2	2	4	5									
4746	0	0	0	0	0	0	0	2	1	2	2	2	2	3	4	3	3	4	4	4	5	4	4
4747	0	0	0	0	0	1	8																
	**ASF-specific clinical sign scores for piglets in litter of inoculated sow 2 (animal ID 4663)**																						
4763	0	0	7	11																			
4764	0	0	0	0	0	1	7	7	7	13													
4765	0	0	0	0	0	0	0	1	1	2													
4766	0	0	0	15																			
4767	0	0	0	1	6	5	7	7	10	9													
4768	0	0	0	2	4	5																	

A grey-coloured box indicates that an animal is not alive on that day

Day post farrowing	D0	D1	D2	D3	D4	D5	D6	D7	D8	D9	D10	D11	D12	D13	D14	D15	D16	D17	D18	D19	D20	D21	D22
Piglet ID	**Rectal temperature of piglets in litter of inoculated sow 1 (animal ID 4662)**																						
4740	38.2	38.2	38.3	38.9	38.8	39.1	39.2	40.4	40.1	41.0	40.5	40.4	39.5	39.6	39.4	39.2	38.9	39.2	39.0	38.9	39.4	39.1	39.2
4741	38.8	38.5	38.6	38.8	39.1	39.1	40.2	40.2	40.5	41.0	41.1												
4742	38.4	39.0	38.5	39.0	39.3	39.6	41.0	41.3	40.2	41.1	40.8	40.4	39.2	39.7	40.0	39.6	39.4	39.7	39.3	38.8	39.6	39.0	39.3
4743	38.9	39.4	39.1	39.3	39.4	40.0	40.5	40.6	41.4	41.6	38.2												
4744	39.1	39.2	38.8	39.3	39.3	40.6	40.9	40.7	40.2	40.7	40.2	40.0	39.6	39.5	39.3	39.4	39.2	39.2	39.4	39.1	39.6	39.3	39.2
4745	38.3	39.0	39.2	39.3	39.4	39.3	39.8	40.4	40.5	40.5	40.9	40.9	40.3	39.6									
4746	37.8	38.5	38.7	39.1	39.4	38.9	39.7	40.4	40.7	40.8	40.9	40.5	40.7	40.4	40.5	39.6	39.5	39.2	39.6	39.5	39.2	39.1	39.6
4747	36.9	38.1	39.1	39.7	39.1	39.5	40.3																
**Average**	**38.3**	**38.7**	**38.8**	**39.2**	**39.2**	**39.5**	**40.2**	**40.6**	**40.5**	**41.0**	**40.4**	**40.4**	**39.9**	**39.8**	**39.8**	**39.5**	**39.3**	**39.3**	**39.3**	**39.1**	**39.5**	**39.1**	**39.3**
	**Rectal temperature of piglets in litter of inoculated sow 2 (animal ID 4663)**																						
4763	38.2	39.2	40.4	41.4																			
4764	38.3	38.9	39.2	39.2	40.4	40.1	41.2	41.0	40.3	39.5													
4765	38.0	39.4	38.6	38.8	39.8	39.9	39.7	40.2	40.5	40.4													
4766	38.5	39.6	39.3	39.9																			
4767	38.4	39.3	40.0	39.9	40.2	39.5	39.9	39.6	40.2	39.6													
4768	38.4	39.5	39.4	40.4	39.9	39.1																	
**Average**	**38.3**	**39.3**	**39.5**	**39.9**	**40.1**	**39.7**	**40.3**	**40.3**	**40.3**	**39.8**													

A grey-coloured box indicates that an animal is not alive on that day

Table S6. Virus load as determined by qPCR (Ct) in blood taken from piglets born from the ASFV-G-ΔI177L-inoculated sows

Day post farrowing	D0	D1	D2	D3	D4	D5	D6	D7	D8	D9	D10	D11	D12	D13	D14	D15	D16	D17	D18	D19	D20	D21	D22	D23	D24	D25
Piglet ID	Virus load in blood (in Ct values) taken from piglets in litter of inoculated sow 1 (animal ID 4662)																									
4740	-	-	-	-	34.5	-	-	-	-	-	-	16.8	-	-	-	-	-	-	19.6	-	-	-	-	-	-	21.9
4741	-	-	-	-	18.7	-	-	-	-	-	15.5															
4742	-	-	-	-	19.1	-	-	-	-	-	-	15.5	-	-	-	-	-	-	17.8	-	-	-	-	-	-	21.2
4743	-	-	-	-	15.5	-	-	-	-	-	17.5															
4744	-	-	-	-	15.4	-	-	-	-	-	-	18.2	-	-	-	-	-	-	19.9	-	-	-	-	-	-	20.9
4745	-	-	-	-	23.9	-	-	-	-	-	-	14.8	-	16.6												
4746	-	-	-	-	14.8	-	-	-	-	-	-	17.7	-	-	-	-	-	-	18.3	-	-	-	-	-	-	19.6
4747	-	-	-	-	14.0	-	15.2																			
4748	32.6																									
4749	36.6																									
4750	34.6																									
4751	37.6																									
4752	30.5																									
4753	27.8																									
4754	45.0																									
4755	18.9																									
4756																										
4757	39.0																									
4758																										
4759																										
4760																										
4761																										
4762																										
	Virus load in blood (in Ct values) taken from piglets in litter of inoculated sow 2 (animal ID 4663)																									
4763	-	-	13.8	-																						
4764	-	-	16.4	-	-	-	-	-	-	17.1																
4765	-	-	20.8	-	-	-	-	-	-	15.6																
4766	-	-	16.0	-																						
4767	-	-	14.0	-	-	-	-	-	-	18.7																
4768	-	-	14.9	-	-	-																				
4769																										
4770																										
4771																										
4772																										
4773																										
4774																										
4775																										
4776																										
4777																										
4778																										
4779																										

dash indicates 'not determined'; a grey-coloured box indicates that an animal is not alive on that day

Table S7. ASF-specific clinical sign scores and virus load as determined by qPCR (Ct) in blood taken from (P5) pigs infected with P4 blood.

Animal Number	Group	Sum of clinical scores per day post inoculation															
		**-1**	**0**	**1**	**2**	**3**	**4**	**5**	**6**	**7**	**8**	**9**	**10**	**11**	**12**	**...**	**28**
2208	A	0	0	0	1	1	1	1	6	6	10						
2209	A	0	0	1	1	2	1	1	7	8							
2210	A	0	0	0	0	1	2	1	6	8	10						
2211	A	0	0	0	0	1	1	1									
2212	A	0	0	0	0	1	2	1									
2213	B	0	0	0	0	1	1	1	1	4	6	13					
2214	B	0	0	0	0	0	2	2	3	5	8						
2215	B	0	0	0	0	0	1	1	n.d.	4	7	14					
2216	B	0	0	0	0	0	2	2	3	5	7	9					
2217	B	0	0	0	0	1	2	1	2	4	6	11					
2218	C	0	0	0	0	0	2	2	1	5	10	18					
2219	C	0	0	0	0	1	1	2	2	6	9	12					
2220	C	0	0	0	0	1	2	1	1	6	7	9					
2221	C	0	0	0	0	0	1	2	2	5	9	20					
2222	C	0	0	0	0	0	1	2	1	4	6	13	16				

a grey-coloured box indicates that an animals is not alive on that day
n.d., not determined

Animal number	Group	Viremia post inoculation (qPCR Ct values)				
		**D0**	**D7**	**D14**	**D21**	**D28**
2208	A	-	12.8			
2209	A	-	13.0			
2210	A	-	12.1			
2211	A	-				
2212	A	-				
2213	B	-	12.2			
2214	B	-	12.2			
2215	B	-	11.9			
2216	B	-	12.8			
2217	B	-	13.5			
2218	C	-	13.3			
2219	C	-	12.6			
2220	C	38.2	13.2			
2221	C	-	12.9			
2222	C	-	12.6			

dash indicates that the qPCR analysis did not generate a signal (no Ct value)
a grey-coloured box indicates that an animal is not alive on that day

Table S8. Variances in poly-C and poly-G stretches of the different isolates.

Samples	14k poly-C (15055-15065)	15k poly-C (16494-16509)	17k poly-G (18450-18464)	17k poly-G (18670-18680)	20k poly-G (20826-20839)
**ASFV-G-ΔI177L inoculum**	11C	16C	15G	11G	14G
**Pregnant sow study, piglet 4745**	11C	17C	17G	11G	14G
**Pregnant sow study, piglet 4765**	11C	18C	14G	11G	14G
***In vivo* passaging, P1 piglet 4644**	11C	16C	15G	11G	14G
***In vivo* passaging, P2 piglet 4647**	12C	15C	15G	11G	14G
***In vivo* passaging, P3 piglet 4648**	12C	15C	14G	11G	14G
***In vivo* passaging, P4 piglet 4650**	12C	15C	14G	11G	14G

Table S9. Higher than 5% ambiguity in the sequences of ASFV-G-ΔI177L-infected piglets.

Piglet no.	ORF	Function of ORF	Localization in the Reference	Potential change	Ratio (%)	Coverage
4745	EP1242L	DNA-dependent RNA polymerase	69356	A->T	64/22	5400
			69361	C->G	72/19	5400
			69372	A-> C	72/24	5100
	C717R	serine/threonine kinases domain	85736	G-> C/T	65/13/18	16800
	B385R	Zinc finger protein, late transcription factor-like	105025	T->G	76/20	18700
			105030	C->T	61/34	18400
			105033	C->T	69/25	18300
4765	EP1242L	DNA-dependent RNA polymerase	69356	A->T	68/20	7200
			69361	C->G	74/21	7200
			69372	A->C	75/21	7000
	C717R	serine/threonine kinases domain	85736	G-> C/T	65/11/18	14500
	B385R	Zinc finger protein, late transcription factor-like	105025	T->G	75/20	18000
			105030	C->T	60/34	17500
			105033	C->T	70/25	17500

Table S10. ASF-related clinical score results of the *in vivo* passage experiment

Passage	Animal number	Just before inoculation										
		Posture	Body shape	Appetite	Activity	Body temperature	Vomiting	Breathing	Neurological signs	Skin	Secretions	Total
1	4644	0	0	0	0	0	0	0	0	0	0	0
	4645	0	0	0	0	0	0	0	0	0	0	0
2	4646	0	0	0	0	0	0	0	0	0	0	0
	4647	0	0	0	0	0	0	0	0	0	0	0
3	4648	0	0	0	0	0	0	0	0	0	0	0
	4649	0	0	0	0	0	0	0	0	0	0	0
4	4650	0	0	0	0	0	0	0	0	0	0	0
	4651	0	0	0	0	0	0	0	0	0	0	0

Passage	Animal number	1 day post inoculation										
		Posture	Body shape	Appetite	Activity	Body temperature	Vomiting	Breathing	Neurological signs	Skin	Secretions	Total
1	4644	0	0	0	0	0	0	0	0	0	0	0
	4645	0	0	0	0	0	0	0	0	0	0	0
2	4646	0	0	0	0	0	0	0	0	0	0	0
	4647	0	0	0	0	0	0	0	0	0	0	0
3	4648	0	0	0	0	0	0	0	0	0	0	0
	4649	0	0	0	0	0	0	0	0	0	0	0
4	4650	0	0	0	0	0	0	0	0	0	0	0
	4651	0	0	0	0	0	0	0	0	0	0	0

Passage	Animal number	2 day post inoculation										
		Posture	Body shape	Appetite	Activity	Body temperature	Vomiting	Breathing	Neurological signs	Skin	Secretions	Total
1	4644	0	0	0	0	0	0	0	0	0	0	0
	4645	0	0	0	0	0	0	0	0	0	0	0
2	4646	0	0	0	0	0	0	0	0	0	0	0
	4647	0	0	0	0	0	0	0	0	0	0	0
3	4648	0	0	0	0	0	0	0	0	0	0	0
	4649	0	0	0	0	0	0	0	0	0	0	0
4	4650	0	0	0	0	0	0	0	0	0	0	0
	4651	0	0	0	0	0	0	0	0	0	0	0

Passage	Animal number	3 day post inoculation										
		Posture	Body shape	Appetite	Activity	Body temperature	Vomiting	Breathing	Neurological signs	Skin	Secretions	Total
1	4644	0	0	0	0	0	0	0	0	0	0	0
	4645	0	0	0	0	0	0	0	0	0	0	0
2	4646	0	0	0	0	0	0	0	0	0	0	0
	4647	0	0	0	0	0	0	0	0	0	0	0
3	4648	0	0	0	0	0	0	0	0	0	0	0
	4649	0	0	0	0	2	0	0	0	0	0	2
4	4650	0	0	0	0	0	0	0	0	0	0	0
	4651	0	0	0	0	0	0	0	0	0	0	0

Passage	Animal number	4 day post inoculation										
		Posture	Body shape	Appetite	Activity	Body temperature	Vomiting	Breathing	Neurological signs	Skin	Secretions	Total
1	4644	0	0	0	0	0	0	0	0	0	0	0
	4645	0	0	0	0	0	0	0	0	0	0	0
2	4646	0	0	0	0	0	0	0	0	0	0	0
	4647	0	0	0	0	0	0	0	0	0	0	0
3	4648	0	0	1	1	2	0	0	0	0	0	4
	4649	0	0	0	0	1	0	0	0	0	0	1
4	4650	0	0	0	0	2	0	0	0	0	0	2
	4651	0	0	0	0	1	0	0	0	0	0	1

Passage	Animal number	5 day post inoculation										
		Posture	Body shape	Appetite	Activity	Body temperature	Vomiting	Breathing	Neurological signs	Skin	Secretions	Total
1	4644	0	0	0	0	0	0	0	0	0	0	0
	4645	0	0	0	0	0	0	0	0	0	0	0
2	4646	0	0	0	0	0	0	0	0	0	0	0
	4647	0	0	0	0	0	0	0	0	0	0	0
3	4648	0	0	1	1	1	0	0	0	0	0	3
	4649	0	0	1	1	1	0	0	0	0	0	3
4	4650	0	0	2	1	1	0	0	0	0	0	4
	4651	0	0	2	1	2	0	0	0	0	0	5

Passage	Animal number	6 day post inoculation										
		Posture	Body shape	Appetite	Activity	Body temperature	Vomiting	Breathing	Neurological signs	Skin	Secretions	Total
1	4644	0	0	0	0	0	0	0	0	0	0	0
	4645	0	0	0	0	0	0	0	0	0	0	0
2	4646	0	0	0	0	0	0	0	0	0	0	0
	4647	0	0	0	0	0	0	0	0	0	0	0
3	4648	0	0	1	1	1	0	0	0	0	0	3
	4649	0	0	1	1	1	0	0	0	0	0	3
4	4650	0	0	1	0	2	0	0	0	0	0	3
	4651	0	1	2	0	2	0	0	0	0	0	5

Passage	Animal number	7 day post inoculation										
		Posture	Body shape	Appetite	Activity	Body temperature	Vomiting	Breathing	Neurological signs	Skin	Secretions	Total
1	4644	0	0	0	0	0	0	0	0	0	0	0
	4645	0	0	0	0	0	0	0	0	0	0	0
2	4646	0	0	0	0	0	0	0	0	0	0	0
	4647	0	0	0	0	0	0	0	0	0	0	0
3	4648	0	1	2	1	2	0	0	0	0	0	6
	4649	0	1	2	1	2	0	0	0	0	0	6
4	4650	0	1	2	1	2	0	0	0	0	0	6
	4651	0	1	2	1	3	0	0	0	0	0	7

Passage	Animal number	8 day post inoculation										
		Posture	Body shape	Appetite	Activity	Body temperature	Vomiting	Breathing	Neurological signs	Skin	Secretions	Total
1	4644	0	0	0	0	0	0	0	0	0	0	0
	4645	0	0	0	0	0	0	0	0	0	0	0
2	4646	0	0	0	0	0	0	0	0	0	0	0
	4647	0	0	0	0	1	0	0	0	0	0	1
3	4648	1	1	2	1	2	0	0	0	0	0	7
	4649	1	1	2	1	2	0	0	0	0	0	7
4	4650											
	4651											

Passage	Animal number	9 day post inoculation										
		Posture	Body shape	Appetite	Activity	Body temperature	Vomiting	Breathing	Neurological signs	Skin	Secretions	Total
1	4644	0	0	0	0	0	0	0	0	0	0	0
	4645	0	0	0	0	0	0	0	0	0	0	0
2	4646	0	0	0	0	0	0	0	0	0	0	0
	4647	0	0	0	0	1	0	0	0	0	0	1
3	4648	2	2	2	2	3	0	0	2	0	1	14
	4649	1	1	2	2	3	0	0	1	0	0	10
4	4650											
	4651											

Passage	Animal number	10 day post inoculation										
		Posture	Body shape	Appetite	Activity	Body temperature	Vomiting	Breathing	Neurological signs	Skin	Secretions	Total
1	4644	0	0	0	0	0	0	0	0	0	0	0
	4645	0	0	0	0	0	0	0	0	0	0	0
2	4646	0	0	0	0	1	0	0	0	0	0	1
	4647	0	1	0	0	1	0	0	0	0	0	2
3	4648											
	4649											
4	4650											
	4651											

0	No abnormalities observed
1, 2 or 3	Abnormality observed
	Humane endpoint

Table S11. Sequence alterations in ASFV-G-I177L during the third and four passages. A, P3 animal 4648; B, P4 animal 4650. The complete sequences were assembled after Illumina sequencing.

Table S11A. The sequence of ASFV in animal 4648 compared to reference ASFV-G-I177L_P0				
**Position in Reference**	**Mutation type**	**Change**	**Affected ORF**	**Change in ORF**
948, 956	Ambiguous	S, W	- (NCR)	-
2210	Insertion	insC (11->12C)	- (NCR)	-
7838	Insertion	insG (5->6G)	- (NCR)	-
15055	Insertion	ins3c (11->14C)	MGF110-11L	ins Gly
16495	Insertion	insC (16->17C)	MGF110-14L	frameshift
18451	Deletion	delG (15->14G)	- (NCR)	-
86868	Point mutation	C->T	C257L	E^{227}->K
104908	Ambiguous	C->Y (C:T=61:39)	B385R	T^{252}->M
192399, 192407	Ambiguous	W, S	- (NCR)	-

Table S11B. The sequence of ASFV in animal 4650 compared to reference ASFV-G-I177L_P0				
**Position in Reference**	**Mutation type**	**Change**	**Affected ORF**	**Change in ORF**
948, 956	Ambiguous	S, W	- (NCR)	-
7838	Insertion	insG (5->6G)	- (NCR)	-
15055	Insertion	ins2C (11->13C)	frameshift	MGF110-11L
18451	Deletion	delG (15->14G)	- (NCR)	
41055	Ambiguous	T->Y (T:C=60:40)	MGF 505-6R	V^{70}->A
86868	Point mutation	C->T	C257L	E^{227}->K
87086	Ambiguous	G->R (G:A=63:37)	C257L	A^{154}->V
87231-87237	Deletion	del3T (7T->4T)	C257L	del K^{105}
192403, 192411	Quasispecies	W, S	- (NCR)	-

Table S12. Mutations during passages in three regions of ASFV-G-I177L. Sequences were obtained by Sanger and/or Illumina sequencing of PCR fragments of P0-P4 viruses.

**Material**	**C257L**	**C257L**	**C257L**	**B385R**	**transgenic region**
	^{86865}ATT**C**CTTG^{86872}	^{87083}CAT**G**CAAC^{87090}	^{87229}CG**TTT**TTTT^{87237}	^{104906}GA**C**GGTC^{104912}	p72-mCherry
**P0**	Cytosine^{86868}	Guanine^{87086}	7 Thymine	Cytosine^{104908}	No nucleotide change
**P1-4644**	C	G	7T	C	
**P2-4647**	Y (C or T)	G	7T	Y (C or T)	
**P3-4648**	Y (C or T)	R (G or A)	7T	Y (C or T)	
**P4-4650**	T	R (G or A)	4T (3T deleted)	C	

Figure S1. The region sequenced with the Sanger methods. The blue boxes highlight the sequenced part of the ASFV genome.

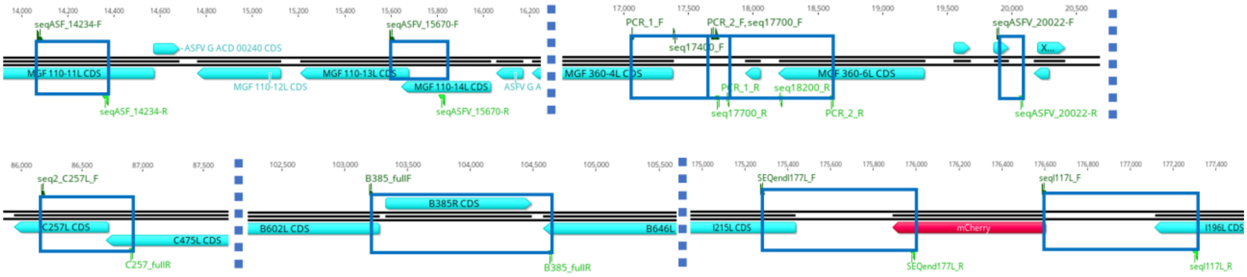

Figure S2. Comparison of the C257L ORF among different ASFV genotypes. The positions that were found to be mutated in the C257L protein of the ASFV genome identified in pig 4650 are amino acid positions 105, 154, and 227. NC_044954, Malawi Lil-20/1, Genotype VIII.; NC_044941, L60, Genotype I.; NC_044945, Ken05/Tk1, Genotype X.; ON409981, TAN/08/Mazimbu, Genotype XV.; ON409980, TAN/16/Magu, Genotype IX.; OR420801, Lisbon 1957, Genotype I.

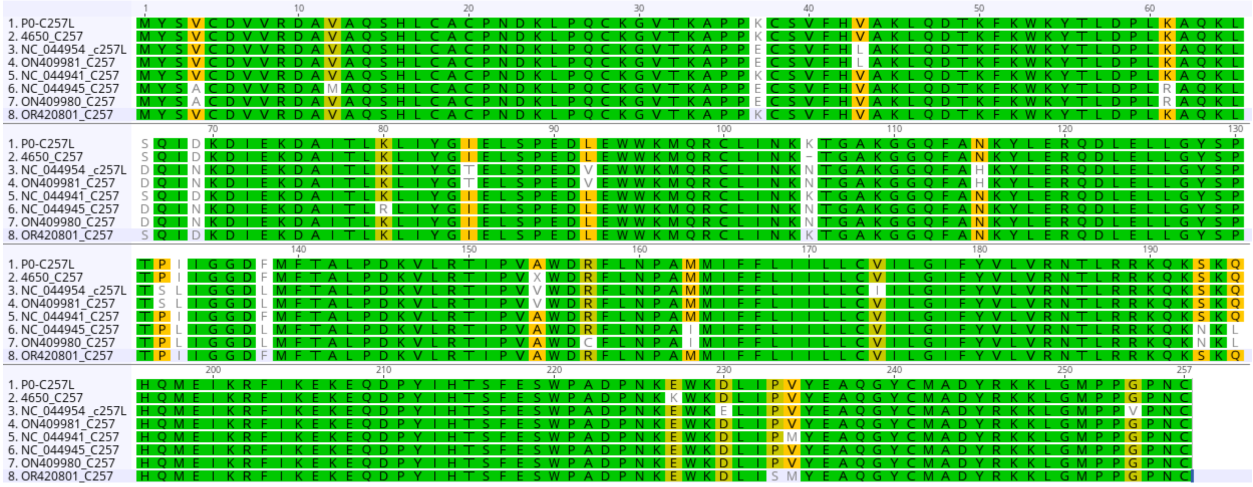

Supplement: Supplementary file 1 — Supplementary datas [file 41541_2025_1099_MOESM1_ESM.pdf]
